# Supplementary material for: Endowing Acceptable Mechanical Properties of Segregated Conductive Polymer Composites with Enhanced Filler-Matrix Interfacial Interactions by Incorporating High Specific Surface Area Nanosized Carbon Black
Source: Nanomaterials (Basel). 2021 Aug 16;11(8):2074. doi: 10.3390/nano11082074 (PMC8400817; doi:10.3390/nano11082074)
Supplement: Supplementary file 1 [file nanomaterials-11-02074-s001.zip › nanomaterials-1323212-supplementary.pdf]

nanomaterials

Supplementary Materials: Endowing Acceptable Mechanical Properties of Segregated Conductive Polymer Composites with Enhanced Filler-Matrix Interfacial Interactions by Incorporating High Specific Surface Area Nanosized Carbon Black

Huibin Cheng [1], Xiaoli Sun [1], Baoquan Huang [1], Liren Xiao [2], Qinghua Chen [1,2,3], Changlin Cao [1,*] and Qingrong Qian [1,2,3,*]

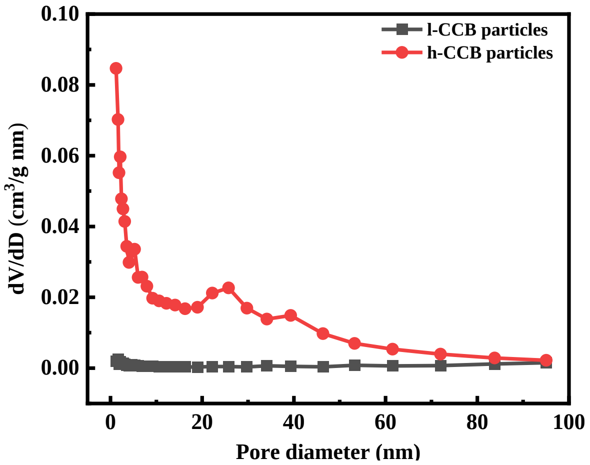

Figure S1. Pore size distributions of h-CCB and l-CCB nanoparticles.

Table S1. Physical properties of two types of conductive carbon black (CCB) nanoparticles.

Sample	S_{BET} (m^2/g)	V_t (cm^3/g)	Pore diameter (nm)
high specific surface area CCB (h-CCB)	380.83	1.7059	17.918
low specific surface area CCB (l-CCB)	8.52	0.0734	34.456

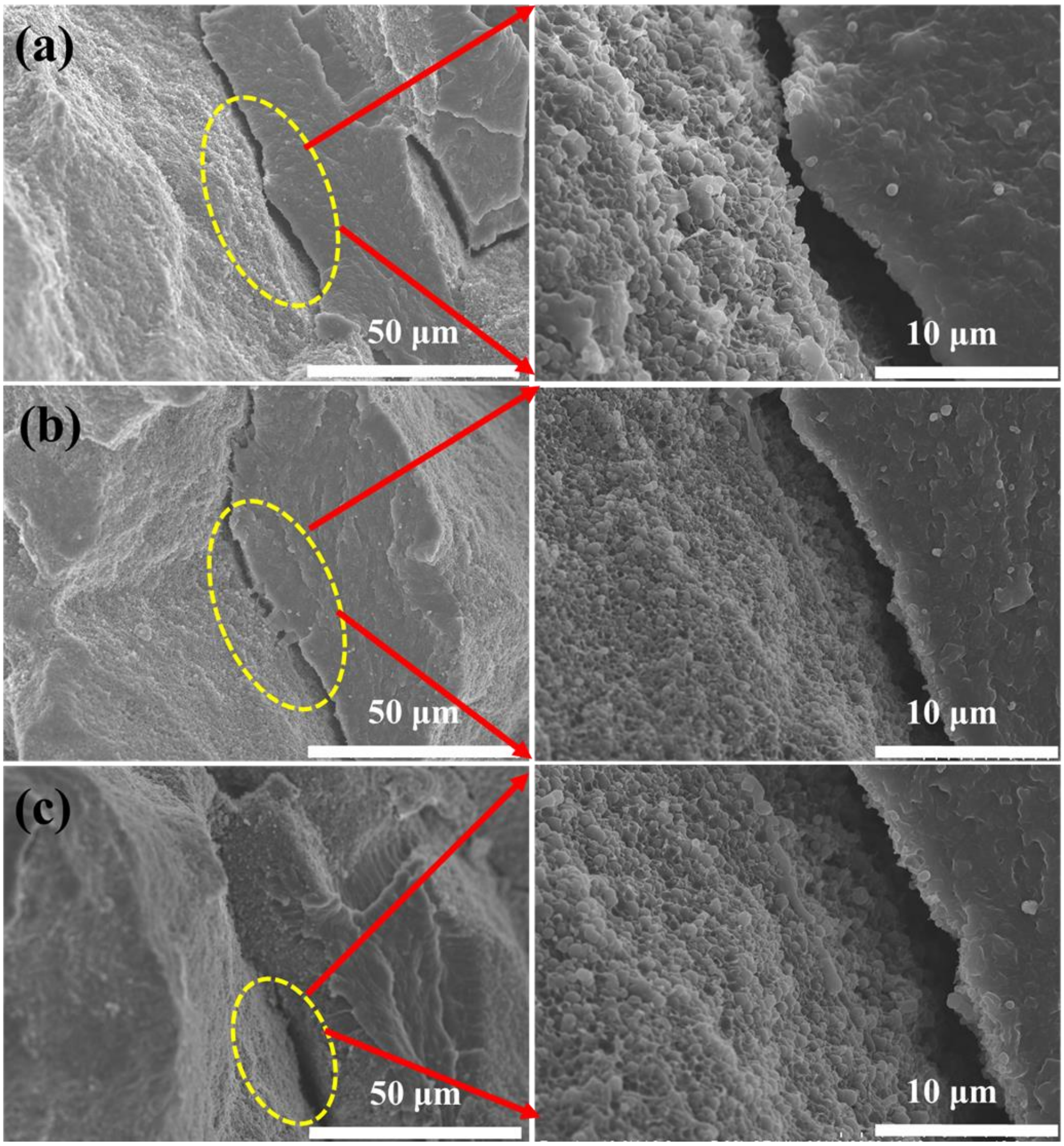

Figure S2. SEM morphologies of quenched section of the segregated UHMWPE composites with the different l-CCB content: (**a**) UHMWPE/l-CCB_5, (**b**) UHMWPE/l-CCB_7, (**c**) UHMWPE/l-CCB_{10}.

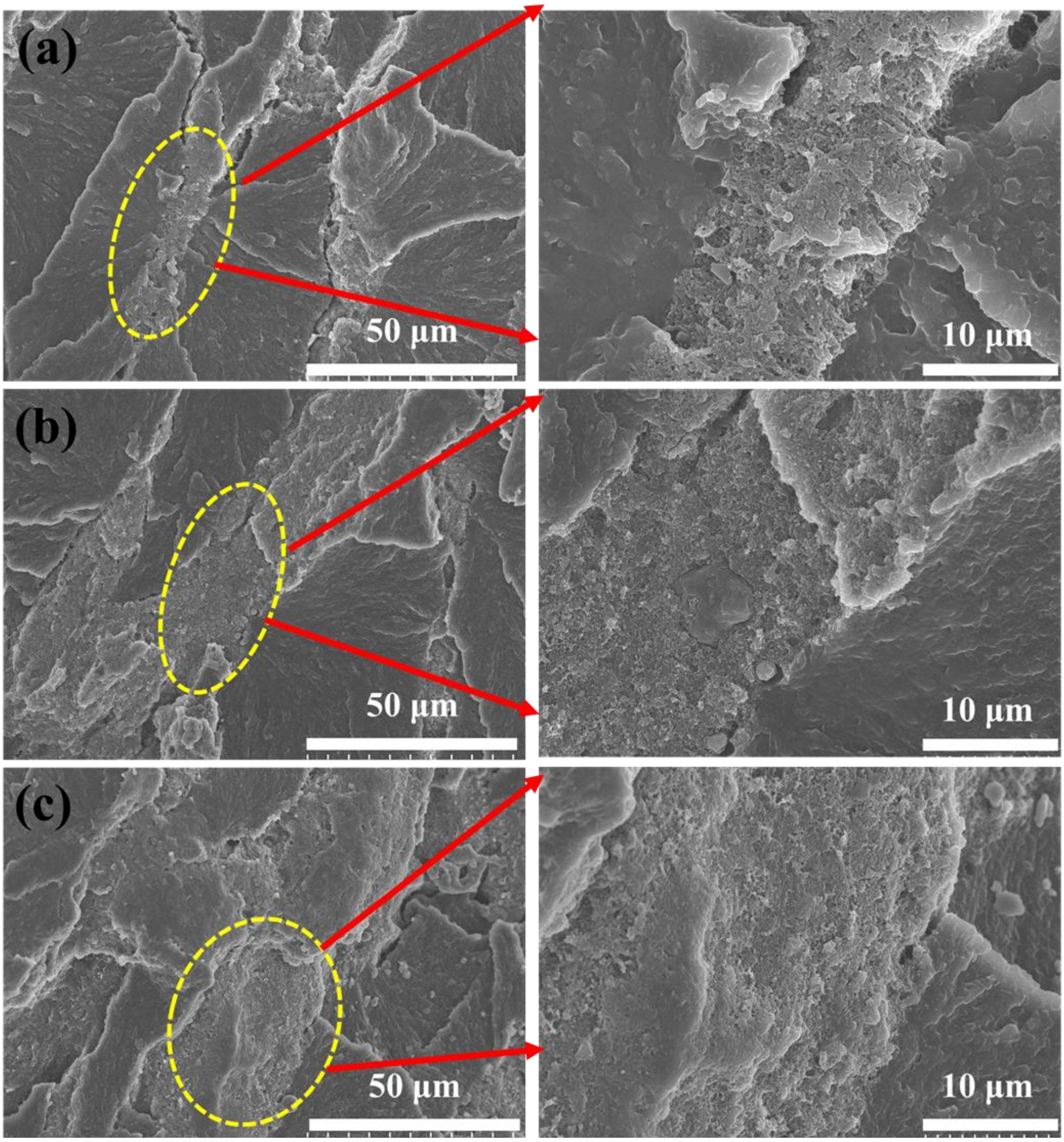

Figure S3. SEM morphologies of quenched section of the segregated UHMWPE composites with the different h-CCB content:(**a**) UHMWPE/h-CCB_5, (**b**) UHMWPE/h-CCB_7, (**c**) UHMWPE/h-CCB_{10}.

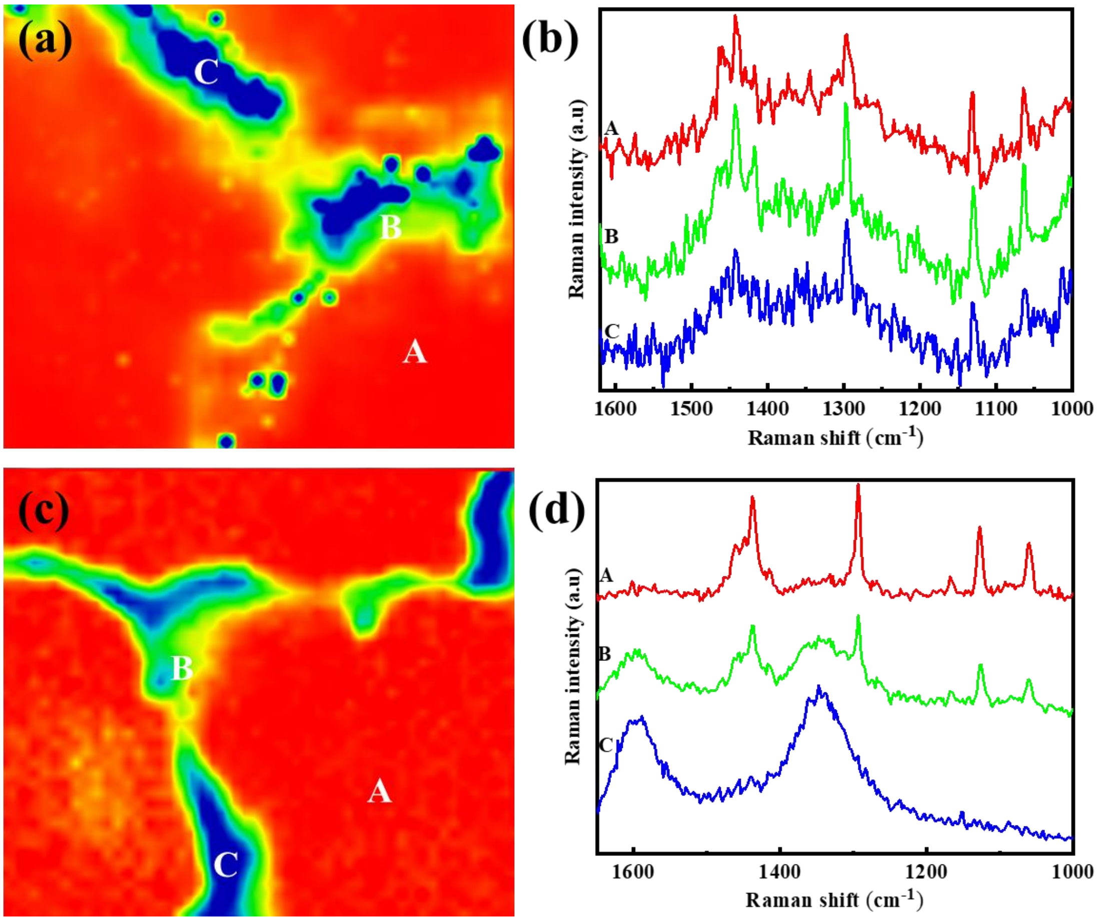

Figure S4. (**a**) Raman mapping images of the UHMWPE/l-CCB0.5 composites, and (**b**) the corresponding Raman spectrum of comparation of UHMWPE/l-CCB0.5 composite at the different position of Raman mapping images, (**c**) Raman mapping images of UHMWPE/h-CCB0.5 composite, and (**d**) the corresponding Raman spectrum of comparation of UHMWPE/h-CCB0.5 composite at the different position of Raman mapping images.

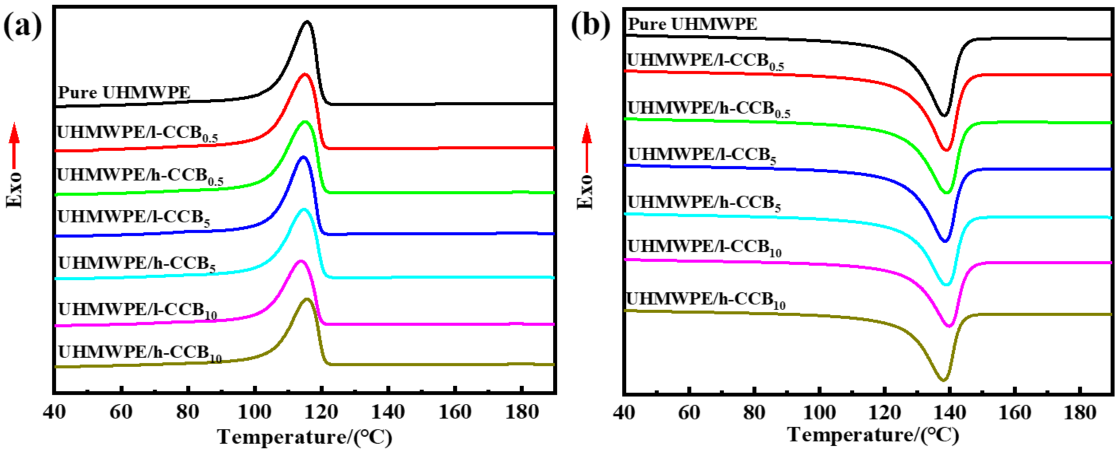

Figure S5. DSC curves of pure UHMWPE, UHMWPE/l-CCB and UHMWPE/h-CCB composites: (**a**) second heating curves; (**b**) cooling curves.

Table S2. DSC values of pure UHMWPE, UHMWPE/l-CCB and UHMWPE/h-CCB composites.

**Samples**	**T_c (°C)**	**$T_{c,onset}$ (°C)**	**T_m (°C)**	**$\triangle H_m$ (J/g)**	**χ_c (%)**
Pure UHMWPE	115.63	121.16	138.37	130.8	44.9%
UHMWPE/l-CCB0.5	114.93	120.73	139.00	129.0	44.5%
UHMWPE/l-CCB1	114.34	119.77	139.94	128.6	44.7%

UHMWPE/l-CCB_3	115.20	120.18	138.85	123.7	43.9%
UHMWPE/l-CCB_5	114.53	120.46	138.62	123.3	44.8%
UHMWPE/l-CCB_{10}	113.84	119.71	139.91	110.7	42.4%
UHMWPE/h-$CCB_{0.5}$	115.01	121.00	139.07	128.8	44.5%
UHMWPE/h-CCB_1	115.32	120.43	138.73	126.4	43.8%
UHMWPE/h-CCB_3	115.16	120.60	138.97	123.1	43.6%
UHMWPE/h-CCB_5	114.78	120.85	139.05	120.9	43.7%
UHMWPE/h-CCB_{10}	115.64	120.71	138.12	110.0	42.0%

The Samples of χ_c is calculated by Equation (S1) [1–2]:

$$\chi C = \frac{\Delta H_m}{(1-\phi)\Delta H_m} \times 100\% \tag{S1}$$

where ϕ is the weight fraction of h-CCB or l-CCB in the composites, ΔH_m is the melting enthalpy of UHMWPE/CCB composites, which was actually determined from the differential scanning calorimetry (DSC) curves, and ΔH_m^o (291 J) is the melting enthalpy of 100% crystallized samples.

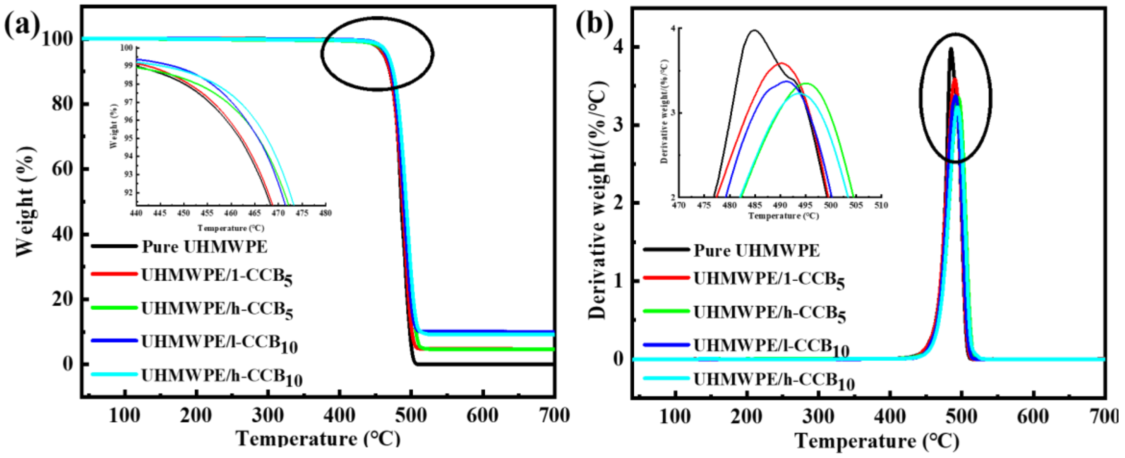

Figure S6. (**a**)TGA and (**b**) DTA curves of UHMWPE, UHMWPE/h-CCB and UHMWPE/l-CCB composites under nitrogen atmosphere.

Table S3. Thermal stabilities of pure UHMWPE, UHMWPE/h-CCB and UHMWPE/l-CCB composites obtained from TGA curves.

**Samples**	**T_5 (°C)**	**T_{30} (°C)**	**T_{50} (°C)**	**T_{max} (°C)**	**$T_{Heat\text{-}resistance\ index}$* (°C)**	**Residue at 700 °C (wt%)**
Pure UHMWPE	453.58	474.50	479.46	478.66	228.40	0.03%
UHMWPE/l-$CCB_{0.5}$	456.85	478.69	485.12	488.62	230.28	0.23%
UHMWPE/l-CCB_1	459.43	480.13	486.09	488.17	231.21	0.63%
UHMWPE/l-CCB_3	462.02	481.49	487.07	487.84	232.11	2.69%
UHMWPE/l-CCB_5	462.31	481.56	487.82	490.21	232.19	4.63%
UHMWPE/l-CCB_{10}	465.70	483.59	489.81	491.18	233.45	9.99%
UHMWPE/h-$CCB_{0.5}$	457.80	476.04	482.08	486.28	229.68	0.22%
UHMWPE/h-CCB_1	456.54	479.72	485.86	488.84	230.52	0.35%
UHMWPE/h-CCB_3	465.47	483.28	489.76	491.77	233.32	2.61%
UHMWPE/h-CCB_5	465.78	485.77	492.52	495.04	234.11	4.56%
UHMWPE/h-CCB_{10}	467.55	486.00	492.65	493.73	234.52	9.17%

The sample's heat-resistance index is calculated by Equation (S2) [3]**:**

$$*T_{Heat\text{-}resistance\ index} = 0.49*[T_5 + 0.6*(T_{30}-T_5)] \tag{S2}$$

In which T_5, T_{30}, and T_{50} are corresponding decomposition temperature of 5%, 30%, and 50% weight loss, respectively.

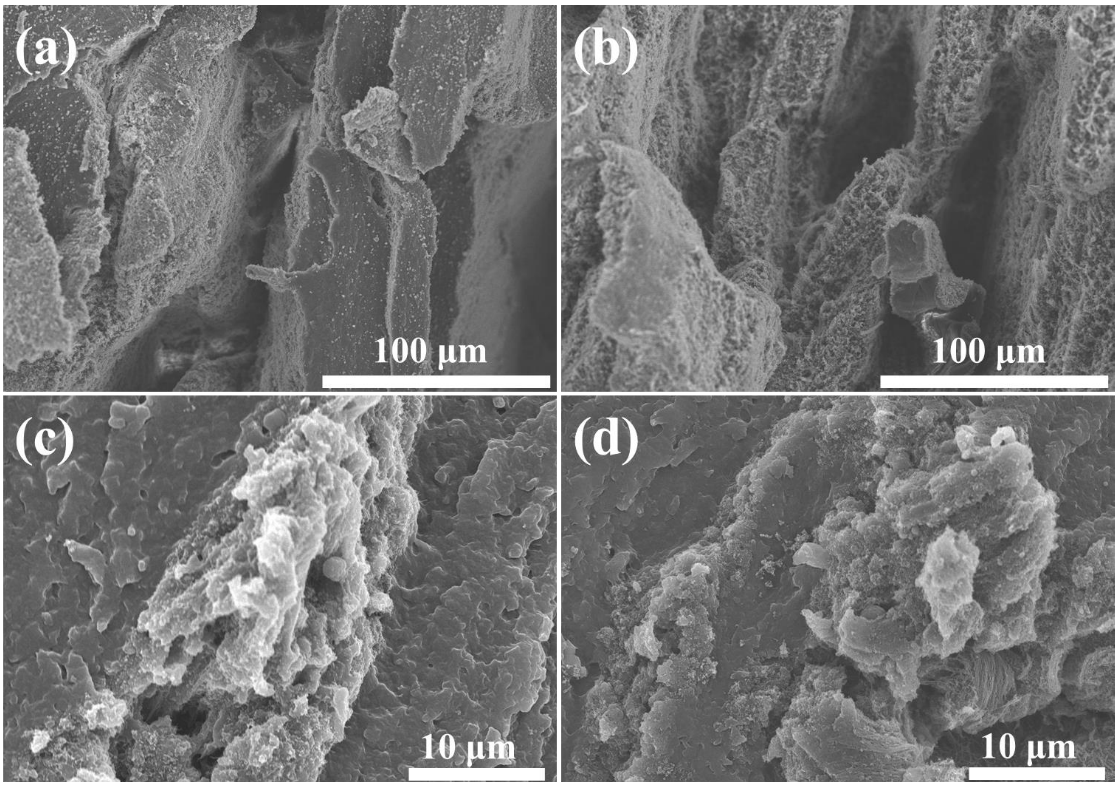

Figure S7. SEM micrographs of the tensile fractured surface of UHMWPE/l-CCB, and UHMWPE/h-CCB composites: (**a**) UHMWPE/l-CCB_{10}, (**b**) UHMWPE/1-CCB_{15}, (**c**) UHMWPE/h-CCB_{10}, (**d**) UHMWPE/h-CCB_{15}.

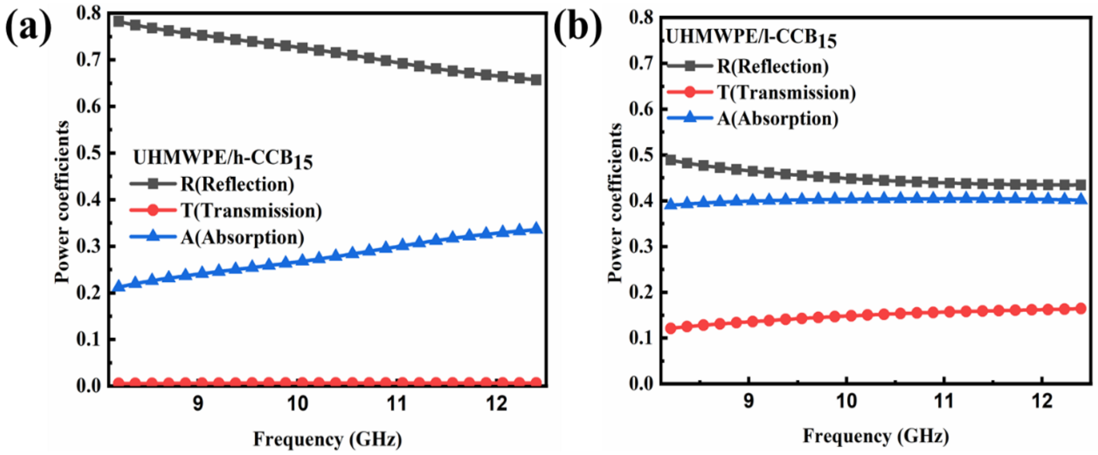

Figure S8. Relationship between power coefficients and frequency of the segregated (**a**) UHMWPE/h-CCB_{15} and (**b**) UHMWPE/l-CCB_{15} composites.

Table S4. comparison of SE_T, SE_R and SE_A at the frequency of 8.2 GHz for UHMWPE/l-CCB and UHMWPE/h-CCB composites.

Sample	SE_A (dB)	SE_R (dB)	SE_T (dB)
UHMWPE/l-CCB_1	0.07	0.26	0.34
UHMWPE/l-CCB_5	1.22	0.67	1.89
UHMWPE/l-CCB_{10}	3.7	1.9	5.6
UHMWPE/l-CCB_{15}	6.25	2.91	9.16
UHMWPE/h-CCB_1	1.91	2.15	3.05
UHMWPE/h-CCB_5	9.65	5.01	14.66
UHMWPE/h-CCB_{10}	15.6	6.70	22.3
UHMWPE/h-CCB_{15}	16.09	6.63	22.72

The obtained scattering parameters were used to calculate EMI SE values [4]:

$$T = |S21|^2 = |S12|^2 \tag{S3}$$

$$R = |S11|^2 = |S22|^2 \tag{S4}$$

$$A + R + T = 1 \tag{S5}$$

$$SE_R = -10\log(1 - R) \tag{S6}$$

$$SE_A = -10\log(T/1 - R) \tag{S7}$$

$$\text{EMI SE} = SE_R + SE_A + SE_M \ (SE_M \text{ can be negligible when } SE \geq 10 \text{ dB}) \tag{S8}$$

References

1. Sun, Z.-F.; Ren, P.-G.; Zhang, Z.-W.; Ren, F. Synergistic effects of conductive carbon nanofillers based on the ultrahigh-molecular-weight polyethylene with uniform and segregated structures. *J. Appl. Polym. Sci.* **2019,** *136*, 47317.
2. Yu, W.-C.; Xu, J.-Z.; Wang, Z.-G.; Huang, Y.-F.; Yin, H.-M.; Xu, L.; Chen, Y.-W.; Yan, D.-X.; Li, Z.-M. Constructing highly oriented segregated structure towards high-strength carbon nanotube/ultrahigh-molecular-weight polyethylene composites for electromagnetic interference shielding. *Compos. A Appl. Sci. Manuf.* **2018,** *110*, 237–245.
3. Guo, Y.; Cao, C.; Cheng, H.; Chen, Q.; Huang, B.; Luo, F.; Qian, Q. Thermal Performances of UHMWPE/BN Composites Obtained from Different Blending Methods. *Adv. Polym. Technol.* **2019,** *2019*, 1–11.
4. Cheng, H.; Cao, C.; Zhang, Q.; Wang, Y.; Liu, Y.; Huang, B.; Sun, X.-L.; Guo, Y.; Xiao, L.; Chen, Q.; Qian, Q. Enhancement of Electromagnetic Interference Shielding Performance and Wear Resistance of the UHMWPE/PP Blend by Constructing a Segregated Hybrid Conductive Carbon Black–Polymer Network. *ACS Omega* **2021,** *6*, 15078–15088.
